# SCANNING FOR CRITICAL EVENTS DURING DRIVING: VISUAL SCANNING USING EYE TRACKING TECHNOLOGY ON A DRIVING SIMULATOR

**DOI:** 10.1093/geroni/igz038.1244

**Published:** 2019-11-08

**Authors:** Anne E Dickerson, Rachel Gartz, Megan Eaker, Brittany Clark

**Affiliations:** 1 East Carolina University, Greenville, North Carolina, United States; 2 Vidant Medical Center, Greenville, North Carolina, United States

## Abstract

This study explored age differences and scanning strategies for critical events using a driving simulator with eye-tracking technology. One critical skill in driving is to efficiently scan the environment. Ten young(M=26y) and 10 old(M=65y) healthy adults drove a wearing Tobii-Pro Glasses. The software analyzes areas of interest (AOI) for “percent time of fixation” as well as illustrative “heat maps” of duration of fixation. At a four-way-stop, the young fixated at the road ahead (25% fixation time), dashboard (29%), more often than left/right of their car (15%). The old fixed on the road ahead (21%) but spent more time outside the car (23%). At the hidden sign, older drivers fixated on the environment outside the road ahead more than younger drivers (26% versus 20%), but both had increased fixation to the right side of the vehicle (Y-23%, O-24%) with this event, likely looking for the sign. Heat maps of the hidden sign illustrate this, suggesting older drivers may more efficiently scan their environment. Other possibilities include the young trusting the simulator, young/old scan differently, or interference of previous simulator experience. Simulator outcomes showed age differences on gas-pedal-reaction times suggesting that older adults are more cautious, slowing down faster with critical events. Limitations include small sample and limited studies have used eye-tracking technology in driving. This study raises interesting questions, especially for medically-at-risk drivers with visual impairments. Using the eye-tracking may enhance targeting specific strategies for a variety of impairments as well as establishing a baseline of typical drivers’ visual scanning habits.

